# An In Vitro Evaluation, on Polyurethane Foam Sheets, of the Insertion Torque (IT) Values, Pull-Out Torque Values, and Resonance Frequency Analysis (RFA) of NanoShort Dental Implants

**DOI:** 10.3390/polym11061020

**Published:** 2019-06-10

**Authors:** Luca Comuzzi, Giovanna Iezzi, Adriano Piattelli, Margherita Tumedei

**Affiliations:** 1 Private practice, via Raffaello 36/a, 31020 San Vendemiano (TV), Italy; luca.comuzzi@gmail.com; 2 Department of Medical, Oral and Biotechnological Sciences, University “G. D’Annunzio” of Chieti-Pescara, Via dei Vestini 31, 66100 Chieti, Italy; gio.iezzi@unich.it (G.I.); apiattelli@unich.it (A.P.); 3 Biomaterials Engineering, Catholic University of San Antonio de Murcia (UCAM), Av. de los Jerónimos, 135, 30107 Guadalupe, Murcia, Spain; 4 Villaserena Foundation for Research, Via Leonardo Petruzzi 42, 65013 Città Sant’Angelo (PE), Italy

**Keywords:** implant stability, insertion torque, pull-out strength, polyurethane foam

## Abstract

Objectives: The aim of this study was to investigate, in polyurethane foam sheets, the primary implant stability of a NanoShort implant compared to a self-condenser implant and to a standard, conventional implant. Materials and Methods: Three implant designs were evaluated in the present in vitro investigation: The Test implant (NanoShort), the Control A implant (self-condenser), and the Control B implant (standard design). The study was conducted by comparing the insertion torque values, the pull-out strength values, and the resonance frequency analysis (RFA) values of the Test and Control A and B implants inserted in polyurethane foam models of different thicknesses and densities. The foam densities were 10, 20, and 30 pounds per cubic foot (pcf). Three thicknesses of polyurethane foams (1, 2, 3 mm) were evaluated for a total of 640 experimental sites. Results: The Pearson correlation showed a moderate/strong correlation between all study groups (r > 0.3) for insertion torque and pull-out strength levels. Increased stability of the Test implants was obtained in 3 mm polyurethane sheets. The 2.5 and 3.5 mm Test implants presented good stability in 3 mm polyurethane sheets of 20–30 pcf densities. The Control implants showed better results compared to the Test implants in 1, 2, and 3 mm polyurethane sheets with densities of 10, 20, and 30 pcf. Conclusions: The NanoShort dental implant evaluated in this in vitro study showed a high level of stability in some experimental conditions, and could represent a useful tool, especially in the posterior mandible, as an alternative to vertical augmentation procedures.

## 1. Introduction

During the insertion of dental implants, bone density plays a key role in determining the primary stability of the implants [1,2]. There is, then, a need to understand the relationship between bone density and primary implant stability to plan implant treatment in a proper way [1]. To get mineralized tissues at the interface with the implants, there is an absolute need to achieve primary implant stability, i.e., the initial biomechanical engagement between bone and implant, with no relative micromovements between these two structures, immediately after insertion of the implants [3,4,5,6]. Poor bone density affects primary stability in a negative way [4,5,7]. Higher bone quality is correlated with higher primary implant stability [6,8]. Therefore, the main factors influencing primary stability are the percentage of bone-to-implant contact (BIC) and the compressive stresses at the implant–bone interface [5]. Besides bone quantity and bone quality, primary stability is also related to implant geometry (length and diameter) and to the surgical technique used to prepare the insertion site of the implants [8]. A low primary stability has been reported to carry a higher risk of implant failure or loss, while with a high stability, better conditions for the formation of mineralized tissues at the implant interface are realized [8]. Moreover, it must also be underlined that a high level of primary stability is associated in a positive way with secondary implant stability [8]. It is, then, extremely important to be able to assess, in an accurate way, primary stability [8].

So far, several non-invasive and non-destructive methods have been suggested to evaluate implant stability, such as insertion torque measurement (IT) and resonance frequency analysis (RFA). IT measures the compression produced by an implant during placement into the surgical site [7] and it is closely related to the primary implant stability, which is considered the most important factor for successful implant treatment [5]. Pull-out torque measures the force needed to remove the implants.

RFA is a validated, useful, and non-invasive tool in the clinical practice [9]. This procedure is able to measure the deflection of the implant–bone complex at different time intervals [10]. Polyurethane foam has been proposed for in vitro tests because it simulates the consistency and density of bone tissue.

The intrinsic mechanical features and biocompatibility of polyurethane foam have been applied in several different medical fields, including vascular engineering and orthopaedics [11,12,13]. This material has been reported to show mechanical properties as described by the ASTM (American Society Testing Materials) F-1839-08 2012 standard [1,4]. Its characteristics render it a very good material to test different implant materials [1], and to standardize the procedures excluding the anatomical and structural differences present in bone [5,6]. The low-to-high densities of polyurethane foams are representative of different bone densities, according to the bone tissue classification D1–D4 proposed by Misch [14].

Short implants (less than 8 mm in length) have been proposed mainly in the clinical condition of deficient alveolar ridges in the posterior jaws [15,16,17]. They could avoid the use of maxillary sinus augmentation procedures or bone grafts, avoiding increased morbidity, higher costs, and higher risks of complications [18,19,20,21]. Several recent systematic reviews with meta-analyses have shown that the survivals of short and standard-length implants are similar [18,19,20,21,22,23]. In the past few years, implants with a reduced length (4, 5, and 6 mm) (Ultrashort or Extrashort implants) have been proposed [16,24]. In the market, it is also possible to find implants with a still-reduced length (2.5 or 3.5 mm) (NanoShort implants) [25].

The aim of the present investigation was to evaluate the in vitro biomechanical behavior of a NanoShort implant compared with a self-condenser implant and a standard implant.

## 2. Materials and Methods

### 2.1. Implants

**Figure 1:**
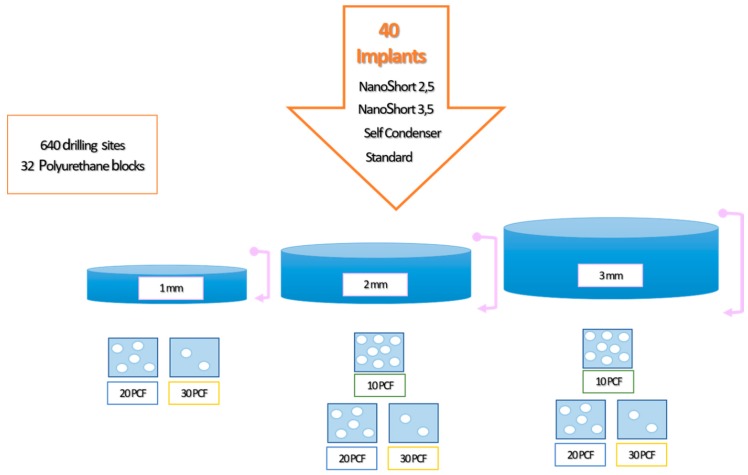
Summary of the model design of the study.

**Figure 2:**
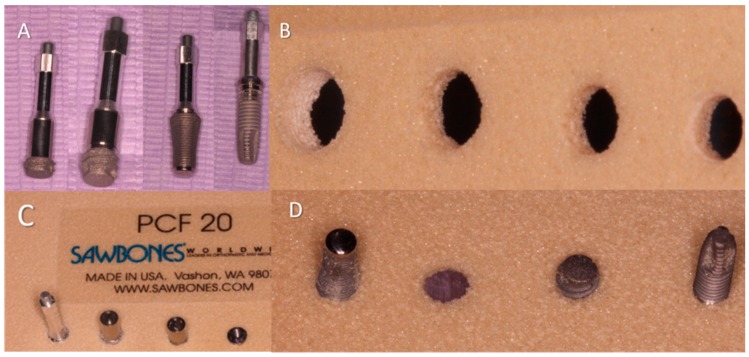
(**A**) From the left to the right: NanoShort 2.5, NanoShort 3.5, self-condenser implant, standard implant; (**B**) details of site preparation of the polyurethane blocks after the drillings protocols; (**C**) implants positioned into a polyurethane block; (**D**) detail of the back view of the positioned implant.

NanoShort titanium dental implants (Oralplant Suisse, Mendrisio, Switzerland; Test Implants) were used for the present in vitro investigation (Figure 1). These implants had a length of 2.5 mm and a diameter of 4.5 mm (Test A), or a length of 3.5 mm and a diameter of 4.1 mm (Test B) (Figure 2A).

SinusPlant^TM^ implants (Oralplant Suisse, Mendrisio, Switzerland) were used as Control A implants, and were 4.5 mm diameter and 10 mm length.

Cylindrical screw-shaped implants were used as Control B implants, and they were of 13 mm length, had a platform diameter of 4.1 mm, and a body diameter of 3.75 mm (Restore, Keystone Dental, Burlington, MA, USA). The coronal portions of two of the experimental implants presented the same diameter, but different shapes: A troncoconical geometry for the Test implants and a cylindrical morphology for the Control B implants (Figure 2A).

### 2.2. Polyurethane Foam Blocks

Polyurethane foam represented a useful tool for biomedical applications, including testing instruments and dental implants for the comparative testing of bone screws [16]. This material has been demonstrated to be able to eliminate the variables of human cadaver bone and of animal bone. Artificial bone blocks presented a uniform cortical bone density and depth, and were unaffected by desiccation. This material exhibited similar properties to bone, and was shown to be reliable and required no special handling or preservation. This synthetic material presented consistent mechanical characteristics.

### 2.3. Experimental Design of the Study

A total of 40 implants (10 Test A, 10 Test B, 10 Control A, and 10 Control B) were used in the present investigation. A total of 640 osteotomies were produced into the polyurethane blocks. Each implant was repositioned 16 times for each experiment, for a total of 10 implants for each study group. The implants were inserted following the protocol of the manufacturer, using an implant lance drill, a 2 mm drill (1600 rpm), and a 3.8 mm final drill (800 rpm) (Figure 1). The handpiece was calibrated at a speed of 70 rpm and a torque of 30 Ncm. Torque values were taken with a software (ImpDat Plus, East Lansing, MI, USA) installed on a digital card. The insertion torque (IT; Ncm) values indicated the force of the maximum clockwise movement that stripped the material. The investigation was conducted by a single operator (LC), comparing the torque insertion and pull-out strength values of the Test, Control A, and Control B implants when inserted into polyurethane foam models of different sizes and densities. Different types of solid rigid polyurethane foam (SawBones H, Pacific Research Laboratories Inc, Vashon, WA, USA) with homogeneous densities were selected for the present investigation. Solid rigid polyurethane foam provided a closed cell content range from 96.0% to 99.9%. Foam was available in a range of sizes and densities, from 0.08 to 0.80 grams per cubic centimeter (5 to 50 pounds per cubic foot). The densities of polyurethane foam used in the present study were 10 pounds per cubic foot (pcf), corresponding to a density of 0.16 g/cm3 (similar to the D3 bone type); 20 pcf, corresponding to 0.32 g/cm3 (similar to the D2 bone type); and 30 pcf, corresponding to 0.48 g/cm3 (similar to D1 bone). Furthermore three different sizes of polyurethane foams were evaluated, for a total of 32 blocks and 640 experimental sites: 13 cm × 18 cm × 1 mm (20, 30 pcf); 13 cm × 18 cm × 2 mm (10, 20, and 30 pcf); and 13 cm × 18 cm × 3 mm (10, 20, and 30 pcf).

### 2.4. Insertion Torque and Pull-Out Torque

The study was conducted by comparing the insertion torque and the pull-out strength values using a calibrated torque meter (UNIKA, Oralplant Suisse, Mendrisio Switzerland) with a torque range of 5–80 Ncm. The final 1 mm insertion torque of the implants into the polyurethane sheets was recorded. In the present study, mechanical torque gauges were used to assess the insertion torque and pull-out strength values. A total of 640 drilling sites (160 for each group) were produced in different sizes and densities of the polyurethane foam models. For each block, 20 drilling sites were obtained.

### 2.5. Implant Drill

Test A implants were inserted following a dedicated drill protocol: A surgical twist drill of 2 mm at 1500 Rpm, a pilot drill of 2–4.5 mm at 800 Rpm, and finally, burs of 4.5 mm diameter and 2.5 mm length.

Test B implants were inserted following a dedicated drill protocol: A surgical twist drill of 2 mm at 1500 Rpm, a pilot drill of 2–4.1 mm at 800 Rpm, and finally, burs of 4.1 mm diameter and 3.5 mm length.

Control A implants were inserted using a surgical twist drill of 2 mm at 1500 Rpm, a pilot drill of 2–3.8 mm at 800 Rpm, osteocondensation screw burs of 10 mm length and 4.5 mm diameter used at the top at 40 Rpm, and then the implant was placed with a predetermined maximum torque of 46 Ncm and 30 Rpm with the surgical motor.

Control B implants were inserted using a surgical twist drill of 2 mm at 1500 Rpm, a pilot drill of 2–3 mm at 800 Rpm, a final 3 mm drill, and then the implant was placed with a calibrated torque of 46 Ncm at a predetermined 30 Rpm with the surgical motor (Figure 2B–D).

### 2.6. Resonance Frequency Analysis

After implant insertion, primary stability was measured using RFA values, expressed using the implant stability quotient (ISQ), with hand-screwed Smart-Pegs (type 7 for Test implants and type 3 for Control implants; Osstell Mentor Device, Integration Diagnostic AB, Savadelen, Sweden). The implant stability quotient (ISQ) ranged from 0 to 100 (measured between 3500 and 85,000 Hz), and was divided into Low (lower than 60 ISQ), Medium (60–70 ISQ), and High stability (more than 70 ISQ) [26]. For each specimen, the RFA measurement was repeated two times. Measurements were performed in two orientations separated by a 90-degree angle, and the average ISQ values were calculated.

### 2.7. Statistical Analysis

Differences between the values of insertion torque, pull-out strength, and RFA of the four groups were analyzed by one-way analysis of variance (ANOVA), followed by Tukey’s post-hoc test. A *p*-value < 0.05 was considered statistically significant. The correlation between the values of insertion torque and pull-out strength was determined by the Pearson Test. Data treatment and statistical analysis were performed by Excel origin and StatPlus6 software.

## 3. Results

**Figure 3:**
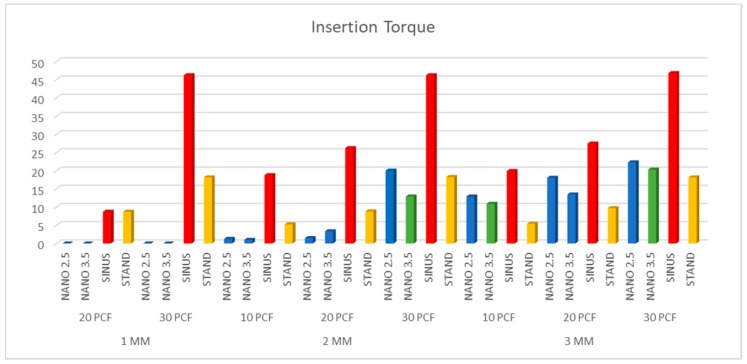
Insertion torque values for the four experimental groups. The self-condenser implant showed the highest ratio of stability.

**Figure 4:**
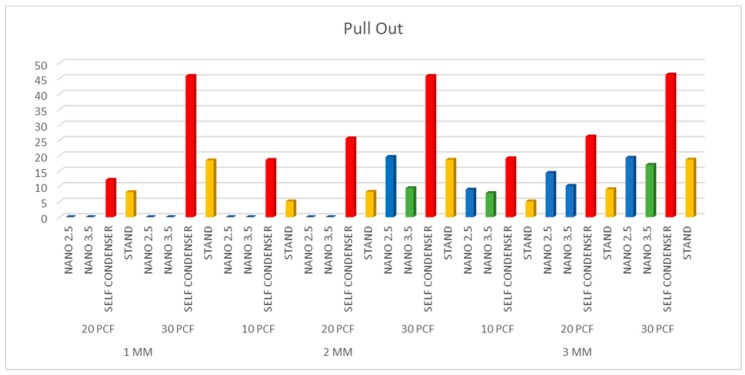
Results of the investigation of pull-out torque. The self-condenser implant showed the highest ratio of stability.

**Figure 5:**
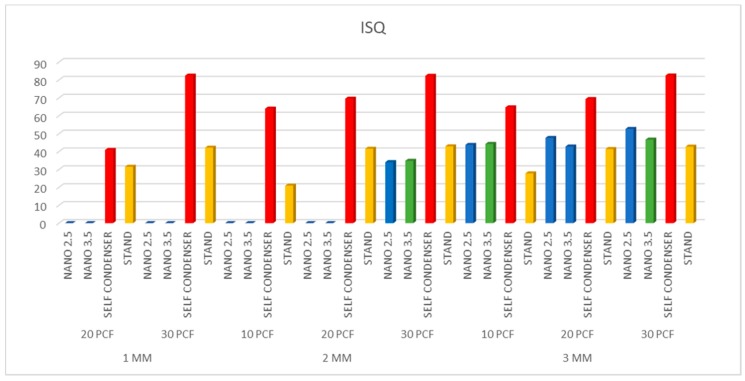
Resonance frequency analysis (RFA) effectiveness of the study groups.

The results were similar when comparing the 2.5 and 3.5 NanoShort implants (Test A and Test B). The Test implants had good stabilization in 3 mm polyurethane sheets of 20–30 pcf densities. In 2 mm sheets, good stability of the Test implants was only obtained in the sheets of 30 pcf density (Table 1). Control A implants showed better results when compared to the Test implants in 1, 2, and 3 mm polyurethane sheets of 10, 20, and 30 pcf densities (*p* < 0.01) (Figure 3, Figure 4 and Figure 5). Control B implants showed good results in 1 and 2 mm polyurethane sheets. In 3 mm sheets, the values of the Control B implants were similar or slightly lower when compared to the Test A and B Implants (*p* > 0.05). The pull-out torque values of the Test implants were lower than the values of insertion torque, while in Control A implants, the values were similar, and in Control B implants, the values of the insertion torque were lower than the values of the pull-out torque (Table 1) (Figure 3, Figure 4 and Figure 5).

## 4. Discussion

Recent systematic reviews with meta-analyses agreed that short implants could be a suitable alternative to more invasive procedures when there is the need to treat alveolar atrophy of the posterior jaws [15,17,18,19,20,21,22,23,24]. Similar survival rates, when compared with standard-length implants, have been reported for short implants. A trend towards a reduction in the implant length of short implants has been observed in recent years, with the introduction in the market of Extrashort and Ultrashort implants (5–6 mm length, or even less) [15,16,17,24]. These implants have been shown to be able to osseointegrate and bear a functional load [24]. Furthermore, Nanoshort implants with a still-reduced implant length (2.5/3.5 mm length) are available in the market [25].

Polyurethane foam could be a useful alternative material to provide mechanical tests for human bone. The American Society for Testing and Materials (ASTM F-1839-08) has approved this material, and has recognized it as a standard for testing instruments and oral implants for the comparative testing of bone screws (“Standard specification for Rigid Polyurethane Foam for Use as a Standard Material for Testing Orthopaedic Devices for Instruments”). Given the difficulties of working with human cadaver bone and animal bone, synthetic polyurethane foams have been widely used as alternative materials in several biomechanical tests, due to the fact that these materials present a similar cellular structure and consistent mechanical characteristics. Artificial bone blocks were chosen over animal or cadaver bone, as they presented a uniform cortical bone density and depth, and were unaffected by desiccation. This material exhibited similar properties to bone, and it was reliable and required no special handling or preservation.

On the contrary, the limitation of this study is that the study design provided only an analysis of the mechanical retention of the implant, whereas in clinical situations, many biological factors affect primary stability, and the physiological and molecular events of the healing of bone tissue produce phenomena like bone resorption, neoformation, and remodeling, leading to secondary stability.

In this way, the reuse of fixture and implant drills did not produce any effect on the outcome of this research, because the study model provided an experimental design that evaluated only the macro-mechanical interactions between the surfaces in the absence of variables of biological interaction that could be conditioned in vivo.

The good primary stability obtained by the Test implants in polyurethane foams of 20 and 30 pcf densities makes them a suitable implant for use in the posterior regions of the mandible, where the bone quality is good and is similar to 30 pcf polyurethane. The polyurethane foam density values of 10–20 pcf are comparable to the D3 bone type. D3 is a bone quality typical of the anterior maxilla and the posterior regions of the jaws. The D3 bone of the anterior maxilla presents less thickness than the mandibular D3.

Yamaguchi et al. (2016) reported that the mean mineral density in the posterior maxilla is 0.31 g/cm. Test implants would probably have less utility in the posterior maxilla, where the bone quality is usually poor. In this anatomic area, Control B implants seem to have higher stability parameters in all the polyurethane heights and densities. The lack of differences between the two different types of Test implants (2.5 vs. 3.5 mm) is probably related to the fact that 2.5 mm implants had a wider diameter (4.5 vs. 4.1 mm), and the PIC (polyurethane-implant contact) was, in all probability, the same. The better results of Control B implants were probably correlated to the fact that these implants were conical, while the Test Implants were cylindrical, and they produced higher friction between the implant and the polyurethane. The similar values obtained by the 2.5 and 3.5 mm implants could probably support the hypothesis that diameter is more important than length [2,7], as the highest load is present in the cortical zone, which is more rigid than the cancellous zone [8], even though some authors have reported that implant length is of great value in obtaining primary stability, particularly in low-density bone [4]. Mohlherich et al. (2015) reported that a significant increase in implant stability was found when there was an increase in bone density [8].

The authors concluded that to achieve the highest possible primary stability, the largest possible diameter must be chosen [8]. Moreover, in cases where it could be possible to use either 2.5 or 3.5 mm Test implants, a preference for the 2.5 mm implants could be suggested due to their easier positioning. When the height of the residual alveolar bone in the posterior mandible is at least 3 mm, it could be preferable to use the Test implants instead of performing a vertical augmentation procedure that has higher morbidity, higher cost, and the need of an additional surgical procedure. The present study showed that in these cases, the Test implants could reach a good stabilization. Furthermore, the use of a higher number of implants could improve the load distribution of the prosthetic restauration. Short, Ultrashort, and NanoShort implants should have an appropriate design to improve the primary stability, even in regions with low bone density [7]. In the case of the Test implants used in the present study, a conical shape would probably have been better than a cylindrical design, and also a different pitch of the threads could have helped to better engage the material. Falco et al. (2018) demonstrated that larger implant threads with a greater pitch could contact more bone trabeculae and better compact the bone debris in a peri-implant location.

## 5. Conclusions

The NanoShort implants used in this study demonstrated an acceptable primary stability in solid rigid polyurethane models when compared to standard implants. The outcome of this investigation suggested a possible clinical application in cases of critical atrophy of posterior mandible, instead of using a more complicated vertical ridge augmentation procedure.

## Figures and Tables

**Table 1:** Values of insertion torque, pull-out strength, and RFA effectiveness of the study groups.

			INSERTION TORQUE		PULL OUT			RFA ANALYSIS	
		Groups	MEAN	SD	MEAN	SD	Pearson Correlation (r)	MEAN	SD
1 MM	20 PCF	NANO 2.5	0	0	0	0	1.000	0	0
		NANO 3.5	0	0	0	0	1.000	0	0
		SELF CONDENSER	14.14	2.049	12.1	1.723	0.69	40.9	1.107
		STAND	10.7	1.625	8.1	1.518	0.76	31.5	1.721
	30 PCF	NANO 2.5	0	0	0	0	1.000	0	0
		NANO 3.5	0	0	0	0	1.000	0	0
		SELF CONDENSER	46.2	1.704	45.8	1.508	0.56	82.43	1.173
		STAND	19.15	1.755	18.4	1.142	0.72	42.15	1.065
2 MM	10 PCF	NANO 2.5	1.25	2.221	0	0	1.000	0	0
		NANO 3.5	1	0.4588	0	0	1.000	0	0
		SELF CONDENSER	19.75	2.049	18.55	1.905	0.75	64	1.564
		STAND	5.25	0.9105	5.1	0.9119	0.34	20.93	2.38
	20 PCF	NANO 2.5	1.45	2.114	0	0	1.000	0	0
		NANO 3.5	3.35	0.7373	0	0	1.000	0	0
		SELF CONDENSER	26.2	1.963	25.55	1.317	0.48	69.58	1.633
		STAND	10.8	1.196	8.2	1.824	0.69	41.58	1.115
	30 PCF	NANO 2.5	20	1.1	19.5	1.433	0.98	34.05	2.299
		NANO 3.5	12.9	0.4224	9.4	2.529	0.61	34.85	2.529
		SELF CONDENSER	46.2	1.704	45.8	1.508	0.36	82.33	1.321
		STAND	19.25	1.803	18.6	1.667	0.80	42.93	1.29
3 MM	10 PCF	NANO 2.5	12.9	2.222	8.95	1.986	0.73	43.75	2.505
		NANO 3.5	10.9	0.2283	7.8	1.262	0.61	44.25	1.262
		SELF CONDENSER	20.9	1.651	19.1	1.518	0.56	64.7	1.712
		STAND	8.4	1.353	5.1	1.373	0.60	27.78	2.835
	20 PCF	NANO 2.5	18.05	2.012	14.35	2.498	0.60	47.65	2.72
		NANO 3.5	13.45	0.3733	10.15	1.928	0.89	42.83	1.928
		SELF CONDENSER	27.45	2.235	26.15	2.007	0.79	69.35	1.702
		STAND	10.7	1.625	9.05	1.849	0.59	41.4	1.283
	30 PCF	NANO 2.5	22.3	2.003	19.25	1.618	0.92	52.68	2.358
		NANO 3.5	20.3	0.3332	16.95	2.047	0.80	46.68	2.047
		SELF CONDENSER	46.8	2.042	46.25	1.832	0.45	82.5	1.225
		STAND	19.15	1.981	18.65	1.814	0.80	42.75	1.323
